# GLP-1 Receptor Agonists: Beyond Their Pancreatic Effects

**DOI:** 10.3389/fendo.2021.721135

**Published:** 2021-08-23

**Authors:** Xin Zhao, Minghe Wang, Zhitong Wen, Zhihong Lu, Lijuan Cui, Chao Fu, Huan Xue, Yunfeng Liu, Yi Zhang

**Affiliations:** ^1^Department of Pharmacology, Shanxi Medical University, Taiyuan, China; ^2^Department of Endocrinology, First Hospital of Shanxi Medical University, Shanxi Medical University, Taiyuan, China

**Keywords:** GLP-1 receptor agonists, endocrine, cardiovascular, neurological, cancer

## Abstract

Glucagon like peptide-1 (GLP-1) is an incretin secretory molecule. GLP-1 receptor agonists (GLP-1RAs) are widely used in the treatment of type 2 diabetes (T2DM) due to their attributes such as body weight loss, protection of islet β cells, promotion of islet β cell proliferation and minimal side effects. Studies have found that GLP-1R is widely distributed on pancreatic and other tissues and has multiple biological effects, such as reducing neuroinflammation, promoting nerve growth, improving heart function, suppressing appetite, delaying gastric emptying, regulating blood lipid metabolism and reducing fat deposition. Moreover, GLP-1RAs have neuroprotective, anti-infectious, cardiovascular protective, and metabolic regulatory effects, exhibiting good application prospects. Growing attention has been paid to the relationship between GLP-1RAs and tumorigenesis, development and prognosis in patient with T2DM. Here, we reviewed the therapeutic effects and possible mechanisms of action of GLP-1RAs in the nervous, cardiovascular, and endocrine systems and their correlation with metabolism, tumours and other diseases.

## Introduction

Glucagon like peptide-1 (GLP-1) is a 30 or 31 amino acid long peptide hormone mainly secreted by 3 tissues in the human body: enteroendocrine L cells in the distal intestine, alpha cells in the pancreas, and the central nervous system (1). Through its interaction with the GLP-1 receptor (GLP-1R), GLP-1 participates in the regulation of glucose homeostasis. In addition, glucagon like peptide-1 receptor agonists (GLP-1RAs) can be combined with GLP-1Rs, playing the same role as GLP-1. A variety of GLP-1RAs and analogues, such as exendin-4 and liraglutide, has been used successfully in the treatment of type 2 diabetes mellitus (T2DM) (2). At present, the therapeutic application and potential value of GLP-1RAs in diseases other than diabetes has become a research hotspot. Interestingly, GLP-1RAs have been reported to activate the metabolism of brown fat and increase the energy expenditure in rodents through exercise activities independent of the sympathetic nervous system pathway (3). Liraglutide has been approved by the United States Food and Drug Administration (FDA) for long-term weight management (4). Moreover, GLP-1RAs have been shown to exert many beneficial effects on vascular endothelial cells. For instance, GLP-1RAs were demonstrated to reduce the risk of cardiovascular events (5) by decreasing the blood pressure (6), improving microvascular function, and reducing inflammation (7). Further, GLP-1RAs play a neuroprotective effect by stimulating the differentiation of nerve cells and inhibiting neuroinflammation (8), while they were also reported to inhibit liver inflammation (9). These findings indicated that in addition to playing a role in the treatment of diabetes, GLP-1RAs can also be used in the treatment of other diseases, such as certain neurological diseases, cardiovascular diseases (CVDs), and diseases related to metabolic disorders. Many studies on the correlation between the function of GLP-1RAs and the development and progression of tumors are also underway. Related studies (10) have found that GLP-1RAs can inhibit the PI3K/AKT/mTOR and ERK/MAPK pathways, thereby inhibiting the growth of prostate cancer; however, whether GLP-1RAs increase the risk of pancreatitis remains controversial (11). We here attempted to systematically review the mechanisms of action and therapeutic value of GLP-1RAs.

## Brief Introduction and Physiological Function of Glucagon Like Peptide-1 Receptor Agonists

Glucagon like peptide-1 (GLP-1) is the second incretin identified in 1983. The first identified incretin was a gastric inhibitory peptide (GIP) with the activity of inhibiting the secretion of gastric acid, which was isolated from porcine small intestines (12). Briefly, GLP-1 exists in the human body in two active forms, GLP-1 (7-36 amide) and GLP-1 (7-37), with the proportion of GLP-1 (7-36 amide) being higher (8, 9). The half-life of natural GLP-1 is very short. Depending on the species, the half-life is approximately 1 to 2 min (13). There are two reasons for this: (1) after its recognition by dipeptidyl-peptidase 4 (DPP-4), GLP-1 is cleaved into GLP-1(9-36) amide, which is in a low affinity; (2) kidney clearance. Glucagon like peptide-1 receptor (GLP-1R) is a member of the B family of G protein-coupled receptors. In the pancreas, the interaction of GLP-1 and GLP-1R is known to mainly act through the cAMP-PKA pathway. More specifically, the interaction of GLP-1 and GLP-1R activates adenylate cyclase (AC), which stimulates the conversion of ATP to cyclic adenosine monophosphate (cAMP), thereby increasing the concentration of cAMP. In turn, cAMP further activates protein kinase A (PKA) and Rap guanine nucleotide exchange factor 4 (RAPGEF4, also known as EPAC2) (14). The activated PKA can close the ATP-dependent K^+^ channel and depolarize the cell membrane, while also activate the voltage-dependent Ca^2+^ channel, causing a Ca^2+^ inflow and the generation of action potentials (15). In addition, PKA can also promote Ca^2+^ release by activating inositol triphosphate (IP3). The activated EPAC2 can further activate Ras protein 1 and phospholipase C, which activate the IP3 and diacylglycerol (DAG) pathways and promote the release of Ca^2+^ (16). All these pathways eventually lead to an increase in the intracellular Ca^2+^ concentration, thereby promoting the mitochondrial synthesis of ATP, and the release of insulin particles into the blood through exocytosis. Apart from the pancreas, GLP-1R is also widely distributed in various tissues of the body including the lungs, kidneys, central nervous system, cardiovascular system, gastrointestinal tract, and skin and vagus nerves (17). This distribution breadth of GLP-1R further highlights the diversity and importance of its biological functions.

**Figure 1:**
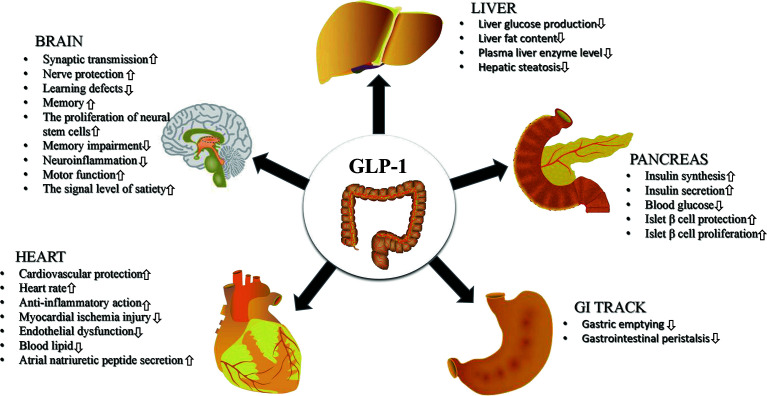
The effects of GLP-1RAs on multiple human organizations. GLP-1RAs exert a positive therapeutic effect on human brain, pancreas, heart, gastrointestinal tract (GI tract) and liver.

Glucagon like peptide-1 receptor agonists (GLP-1RAs) are emerging glucose control drugs, which are widely used in the treatment of T2MD in recent years. Due to the wide distribution of GLP-1R, GLP-1RAs also have a wide range of pharmacological effects. At present, existing GLP-1RAs mainly include 2 types: polypeptide and non-polypeptide (18). Based on similarities in their amino acid sequence, peptide agonists are mainly divided into GLP-1 and derivatives and exendin-4 and derivatives. Common GLP-1RAs are listed in **Table 1**. In addition to lowering the levels of blood glucose, as shown in **Figure 1**, GLP-1RAs have also been shown to have a positive effect on multiple human tissues. Liraglutide, which has been approved for reducing the risk of T2MD, has also been found to reduce the risk of major cardiovascular events in adults with established CVDs (19). Accordingly, the guidelines of the American Diabetes Association recommend it as a second-line drug after metformin, suitable for patients with known atherosclerotic cardiovascular disease (20). Moreover, liraglutide was reported to reduce the abnormal proliferation of hyperglycaemia-induced vascular smooth muscle cells by inhibiting the PI3K/AKT and ERK 1/2 signaling pathways (21). Due to the metabolic regulatory function of GLP-1RAs, they have also been considered for the treatment of other diseases such as obesity, liver disease and other metabolic dysfunction diseases (22).

**Table 1:** Classification of GLP-1RAs.

Name	Listing situation	Molecular formula	Molecular weight
Exenatide	Yes	C_149_H_234_N_40_O_47_S	3369.76000
Liraglutide	Yes	C_172_H_265_N_43_O_5_1	3751. 20Da
Lixisenatide	Yes	C_215_H_347_N_61_O_65_S	4858.50000
Albiglutide	Yes	C_148_H_224_N_40_O_45_	3283.65000
Dulaglutide	Yes	C_2646_H_4044_N_704_O_836_S_18_	59669.00000
Semaglutide	Yes	C_187_H_291_N_45_O_59_	4113.57754
Beinaglutide	Yes	C_149_H_225_N_39_O_46_	3298.7Da
Supaglutide	Clinical Trials	Waiting to be published	Waiting to be published
PEG-loxenatide	Yes	C_210_H_325_N_55_O_69_S·(C_2_H_4_O)_2n_	44212.65 ± 4000Da

In non-alcoholic fatty liver disease (NAFLD) mice (23), liraglutide was shown to inhibit the MKK4/JNK signaling pathway, thereby improving the hypoadiponectinemia-induced inflammatory stress. In addition, GLP-1RAs (24) have also been found to play an active role in the treatment of polycystic ovary syndrome (PCOS). Besides, the protective and therapeutic effects of GLP-1RAs on the central nervous system have now been confirmed by several studies (25, 26). In particular, they have been reported to stimulate nerve differentiation, enhance synaptic plasticity, promote nerve cell survival, and prevent and treat Alzheimer’s disease (AD), Parkinson’s disease (PD), stroke and other neurological diseases by regulating the expression of some key enzymes and the release of certain neurotransmitters (27). In addition, a number of studies have also shown that GLP-1RAs has a positive effect on the treatment of certain tumors (10).

## Effects of Glucagon Like Peptide-1 Receptor Agonists on the Neurological System

Autoradiography of human tissue sections with ^125^I-labelled GLP-1 (7-36 amide) revealed a high content of GLP-1R in the central nervous system suggesting its potentially important role in the nervous system (28). More specifically, GLP-1R is widely expressed in the periphery of the central nervous system, including the hippocampus, neocortex, hypothalamus, spinal cord and cerebellum (29). In recent years, a variety of mechanisms for the beneficial effects of GLP-1RAs on the brain have been discovered, including the reduction of neuroinflammation and increase of signal transduction in surviving cells. In addition, they have been shown to enhance synaptic transmission and counteract learning deficits (30, 31). A recent study found that exendin-4 could improve the reference memory ability of adult rats (32). The use of GLP-1RAs could promote the proliferation of adult rodent neural stem cells, indicating their role in promoting brain regeneration (33, 34). The therapeutic effect of GLP-1RAs on neurological diseases has been confirmed by several studies.

## Effects on Alzheimer’s Disease

Alzheimer’s disease (AD) is a progressive and irreversible neurodegenerative disease, with unclear aetiology and pathogenesis. Approximately 6-8% of people over the age of 65 suffer from AD; this risk is known to increase with age, and is more common in women (1.5 times compared with men) (35). A study has suggested that the accumulation of amyloid-β (Aβ) deposits triggers a series of pathological processes, such as inflammation, tau angle formation, synaptic dysfunction, and cell death, which lead to neurodegenerative behavior and eventually to the development of dementia (36). According to some researchers, the abnormal accumulation of Aβ and tau protein is the cause of AD (37, 38). However, some studies have indicated that these are manifestations and not the cause of the disease (39, 40). The main pathological features of AD include the deposition of insoluble Aβ that forms senile plaques, neurofibrillary tangles, and neuronal apoptosis (40). Neurofibrillary tangles have been found in the amygdala, hippocampal structure, parahippocampal gyrus, and temporal cortex of patients with AD, whereas senile plaques have been shown to be distributed throughout the combined neocortex and striatum (41). Interestingly, AD has been associated with a dysfunction in insulin signaling in the brain. A Rotterdam study has shown that the risk of AD in patients with T2MD is increased by 2-fold (42). Moreover, insulin resistance(IR) has also been reported in the brain of patients with AD. Due to IR, the brain cannot use glucose, thus leading to inflammation and the deposition of plaques and tangles (43).

In an AD mouse model, GLP-1RAs were demonstrated to reduce the levels of AD pathological markers, including oligomeric antibodies and antibody plaque load, reduce the activation of microglia, and improve memory behavior (35, 44, 45). Furthermore, GLP-1RAs were found to protect hippocampal neurons from cell necrosis caused by glutamate, Fe^2+^ and hypoxia. Calcium is also known to play an important role in neuronal plasticity and neurodegenerative diseases. In a study using cultured rat hippocampal neurons, pre-treatment of nerve cells with GLP-1 resulted in a weakened Ca^2+^ response to glutamate and weakened membrane depolarization (46). Whole-cell patch clamp analysis showed that glutamate-induced currents and voltage-dependent calcium channel currents were significantly reduced in GLP-1-pre-treated neurons. Pre-treatment of neurons with GLP-1 significantly reduced the susceptibility of glutamate-induced neuronal death. A basic study suggested that semaglutide protects neurons from Aβ toxicity potentially through the enhancement of autophagy and the inhibition of apoptosis (47). Zhou et al. found that the protective effect of dulaglutide on learning and memory impairment might be the result of reducing the hyperphosphorylation of tau and neurofilament proteins in a PI3K/AKT/GSK3β signaling pathway-dependent manner (48). Two types of GLP-1RAs, liraglutide and exenatide, were found to be antagonistic to the neurodegeneration and AD progression even in mice without diabetes (35, 49). McClean et al. (35) showed that systemic administration of liraglutide in AD transgenic mice for 8 weeks could prevent memory impairment, neuronal loss, and deterioration of hippocampal synaptic plasticity. In addition, according to the numbers of activated microglia, liraglutide could significantly reduce both the deposition of amyloid plaques and inflammation (50). Similarly, in rats injected with monoclonal antibodies (mAbs) in the hippocampus, pre-treatment with liraglutide significantly protected from the mAbs-induced damage of spatial memory and long-term potentiation (51). Importantly, liraglutide was not only shown to have a preventive effect, but also reversed some of the key pathological features of late AD in mice (52). Another GLP-1 analogue, exenatide, has also shown promising results against neurodegenerative diseases in a pre-clinical study (53). Conclusively, GLP-1RAs have great research potential in the field of AD treatment.

## Effects on Parkinson’s Disease

Parkinson’s disease (PD) is a chronic neurodegenerative disease that affects the central nervous system and the second most common neurodegenerative disease after AD (54). It mainly affects the motor nervous system, and is associated with the loss of Lewy bodies and substantia nigra dopamine neurons (55). The Lewy body is a neuron inclusion body, mainly composed of α-synuclein (56). Dopamine, which acts as a neurotransmitter, is known to play a key role in movement control. However, the reason behind the loss of these dopamine-producing nerve cells remains unclear (57). The most obvious symptoms of PD in the early stages are tremor, limb stiffness, decreased motor function and abnormal gait. Cognitive and behavioral problems might also manifest. In the late stages of PD, the key molecular pathogenic mechanisms include the misfolding and aggregation of α-synuclein, mitochondrial dysfunction, impaired protein clearance (related to inefficient ubiquitin-proteasome and autophagy-lysosomal systems), neuroinflammation and oxidative stress (57). To date, no therapeutic approach has been shown to either completely cure PD or delay, stop, and reverse the degeneration and death of dopaminergic neurons (58). Therefore, effective neuroprotective therapies are continuously being pursued. Increasing evidence has demonstrated that GLP-1 analogues can cross the blood-brain barrier, protect dopaminergic neurons in the substantia nigra, and rescue motor activity and cognitive functions in PD animal models (59–61). These findings have strongly supported the hypothesis that the use of GLP-1RAs might be a novel effective treatment for PD.

Disturbance of insulin signaling in diabetic patients might lead to the abnormal expression of αSyn, damage to mitochondrial function, increase in mitochondrial oxidative stress, and down-regulation of the PI3K/AKT pathway, which in turn could promote the occurrence and development of PD (62). Surprisingly, GLP-1RAs, such as liraglutide, lixisenatide, and semaglutide, have showed outstandingly neuroprotective effects on animal models of PD (63, 64). Bertilsson et al. found that intraperitoneal injection of exendin-4 increased the number of BrdU-positive progenitor cells in the subventricular zone (33), indicating that exendin-4 could compensate for the loss of dopaminergic neurons in the PD model by promoting the formation of substantia nigra neurons. Similarly, in a 1-methyl-4-phenyl-1,2,3,6-tetrahydropyridine (MPTP)-induced PD mouse model, GLP-1 analogues were reported to protect the brain from MPTP-induced pathological effects, such as movement disorders, increased levels of α-synuclein, chronic inflammation in the brain, loss of dopaminergic neurons, oxidative stress, and expression of growth factors (65). The novel GLP-1RA geniposide was found to up-regulate the expression of β-cell lymphoma 2 (Bcl-2), whereas reduce the activity of caspase 3, thereby protecting dopaminergic neurons in a MPTP mouse model of PD (66). Inflammation is increasingly recognized as a key factor in the pathogenesis of PD (67). Therefore, the regulation of the activity of microglia is believed to play a key role in the neuroinflammation of PD. Positron emission tomography of patients with early PD showed a significant increase in the activation of microglia (68), which might lead to neuron loss in PD and AD (69).

Injection of exendin-4 into rodents after endotoxin and mTP toxin-induced damage to the substantia nigra striatum could prevent the toxin-induced activation of microglia and inhibit the production of pro-inflammatory cytokines (70). Mitochondrial function interference or mitochondrial damage is also one of the mechanisms of pathogenesis of PD (71). Interestingly, GLP-1RAs have also been shown to exert multiple effects on mitochondria. For example, saxagliptin protected the mitochondrial function in PD rat models by up-regulating complex I and the anti-apoptotic protein Bcl-2 (72), while long-term use of exendin-4 in rodents promoted the function of hippocampal nerves, and improved motor function and behavior (73). Moreover, GLP-1RAs were shown to protect neurons and induce beneficial changes in neuroplasticity in laboratory models of several neurological diseases, including PD (31). In a pre-clinical trial, patients with moderate PD who received subcutaneous injection of 2 mg exenatide once a week had an advantage of 3.5 points in the mds-updrs exercise scale over the placebo group (74). Zhang et al. found that semaglutide could reverse the decrease in the levels of tyrosine hydroxylase, alleviate inflammation, and increase autophagy, thus protecting dopaminergic neurons in substantia nigra and striatum (75). Another study showed that the incidence of PD diagnosed with T2DM varies greatly depending on the administered diabetes treatment. Compared with other oral hypoglycemic agents, the prevalence of PD when using DPP4 inhibitors and GLP-1RAs was shown to be 36-60% lower (76). These results indicated that GLP-1RAs might play a useful role in the future treatment of PD.

## Effects on Stroke

Stroke is the second leading cause of death and the leading cause of disability worldwide. Strokes are mainly divided into hemorrhagic and ischemic strokes caused by blood vessel obstruction. Strokes are mostly caused by arterial occlusion, which in turn leads to cerebral ischemia, brain damage, and then nerve damage and disability. Typical symptoms of stroke include sudden unilateral weakness, numbness or loss of vision, diplopia, changes in speech, ataxia, and non-orthogonal vertigo (77). Atypical symptoms include isolated vertigo, blindness in both eyes, amnesia, agnosia, dysarthria, dysphagia, stridor, headache, hemiplegia, confusion, and changes in consciousness (77). The pathological mechanism of stroke mainly includes the apoptosis of neurons in the cerebral cortex and striatum. As neurological and medical complications after stroke are not properly predicted, prevented, or dealt with, they are considered to constitute the main cause of the high morbidity and mortality associated with strokes (78). In addition, T2DM is associated with an increased risk of stroke and mortality after stroke (79). Interestingly, GLP-1RA antidiabetic drugs have shown a distinct effect in reducing the incidence of stroke and enhancing neuroprotection in both pre-clinical and clinical studies (80, 81).

A pre-clinical study showed that exendin-4 had a remarkable neuroprotective effect, improving the neurological deficit caused by transient middle cerebral artery occlusion in mice, and reducing neuronal loss and microglial inflammation (82). Acute administration of exendin-4 at the beginning of the stroke or 1 h later was reported to have a significant effect; however, this neuroprotective effect disappeared after 3 h. The neuroprotective effect of exendin-4 was found to be independent of the glucose-increasing effect, whereas related to increasing the levels of cAMP and reducing oxidative stress and the inflammatory response (83). Relevant studies have found that exendin-4 could mediate the neuroprotective effect on γ-aminobutyric acid neurons in the piriform cortex and striatum and play a neuroprotective role in a cAMP/PKA- and PI3K/AKT-dependent manner after stroke (84, 85). Sato et al. found that intraperitoneal injection of liraglutide 2.5 h after stroke induced neuroprotection in rats, which was related to the up-regulation of vascular endothelial growth factor (VEGF) (86). A study on the protective mechanism of liraglutide on cortical neurons after ischemia suggested that liraglutide probably reversed the ischemia-induced apoptosis by activating the PI3K/AKT and MAPK pathways (87). Another study on the effect of dulaglutide on stroke showed that dulaglutide reduced the incidence of stroke in middle-aged and elderly people with T2MD and other cardiovascular risk factors, but it cannot reduce the severity of stroke (88). A trial of reperfusion therapy for ischemic stroke indicated that semaglutide, which has a strong GLP-1R-mediated neuroprotective effect, could reduce the infarct size in acute ischemic stroke in non-diabetic rats (89). In addition, the activation of GLP-1R was shown to promote synaptic plasticity and axonal growth (30), and stimulate adult neurogenesis (90). These findings might lay the foundation for the potential regenerative treatment of patients with chronic stroke.

## Effects on Chronic Pain

Pain is an unpleasant subjective and emotional experience related to actual or potential tissue damage (91). Pain can be divided into acute and chronic pain (92). Compared with acute pain, chronic pain is known to be much more harmful to the human body (93). Continuous pain can induce more serious pathological reactions in the human body, and even cause shock or death. Therefore, the treatment of chronic pain is even more important. Clinically, treatment drugs for chronic pain are mainly non-steroidal anti-inflammatory analgesics, opioid analgesics, and auxiliary drugs: such as antidepressants and anxiety drugs (94). However, the adverse reactions caused by these drugs often limit their application. In particular, long-term use of non-steroidal anti-inflammatory drugs (NSAIDs) can cause gastric mucosal damage as they can inhibit mitochondrially encoded cytochrome C oxidase I (MT-CO1, also known as COX-1) and block its protective effect on gastric mucosa. Therefore, the use of NSAIDs is usually associated with damage to the gastrointestinal tract and other systems during treatment, and thus need to be used with caution (95). In recent years, there have been numerous crises caused by the abuse of opioids. The nervous system can quickly develop tolerance to the drug. Overuse of opioids might lead to serious consequences, such as respiratory depression, addiction, and even death (96, 97). Studies found that intrathecal injection of GLP-1RAs effectively reduced formalin-induced peripheral nerve injury, as well as cancer- and diabetes-induced pain without causing serious adverse reactions. Moreover, it was also reported that long-term injection of geniposide and exenatide did not induce nociceptive tolerance (98, 99). Hence, GLP-1RAs (98) appear to be potential alternatives for the treatment of chronic pain due to their effect in reducing pain while not affecting acute injury. Previous experiments have found that exendin-4 could reduce the pain-induced neuro-inflammatory response through the GLP-1R pathway, thereby promoting the recovery of cognitive function in mice (100). Likewise, the anti-dipeptidyl peptidase-IV GLP-1RA ROSE-010 was shown to effectively relieve the intestinal pain induced by irritable bowel syndrome (101). However, there have been relatively few studies on the mechanism of GLP-1RAs in the treatment of chronic pain.

**Figure 2:**
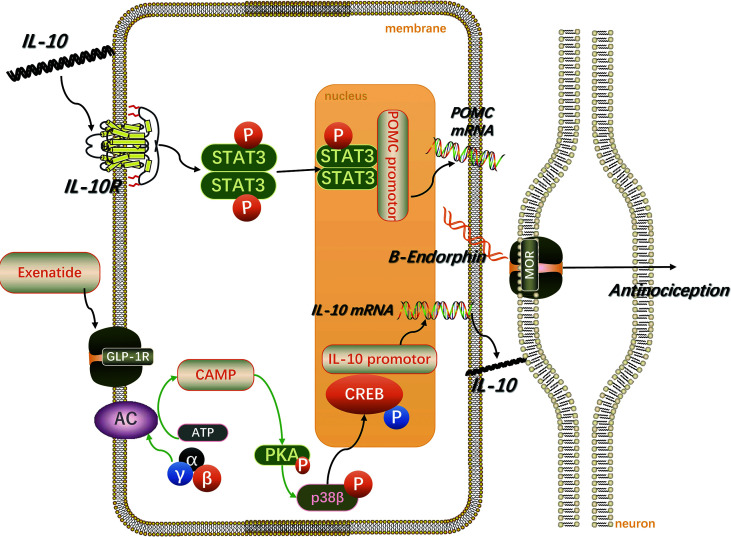
Spinal microglia GLP-1R/IL-10/β-endorphin analgesic pathway. IL-10 stimulates the GLP-1R activation-induced microglial expression of β-endorphin and neuropathic spinal cord anti-allergic response in an autocrine manner. The figure is modified from Ref 103.

Surprisingly, a recent study found that liraglutide blocked the lipopolysaccharide-induced visceral allodynia in a NO-dependent manner. It was suggested that this was achieved by inhibiting the production of pro-inflammatory cytokines and reducing the increase in intestinal permeability (102). As shown in **Figure 2**, following stimulation of GLP-1R by exenatide on microglia, the cAMP/PKA/p38β/CREB signal transduction pathway is activated, promoting the expression of IL-10. Subsequently, the IL-10 receptor/STAT3 signaling pathway in microglia is autocrinally activated, thereby promoting the expression and release of β-endorphin, the latter acts on μ-opioid receptors on neurons to produce analgesic and neuroprotective effects (103–105).

The various aforementioned GLP-1RAs are peptides that are easily hydrolyzed by proteases, thus making their oral use basically ineffective. Therefore, discovering a small molecule GLP-1RA that could be orally administered is a current but extremely challenging research hotspot. Interestingly, W-24 (106), a non-peptide GLP-1RA, has been reported to inhibit inflammatory pain by stimulating the spinal cord GLP-1R for the release of analgesic β-endorphin. Moreover, as a small molecule drug, it could increase the confidence of in-depth research to find oral GLP-1RAs in the treatment of chronic pain.

## Effects of Glucagon Like Peptide-1 Receptor Agonists on the Cardiovascular System

**Figure 3:**
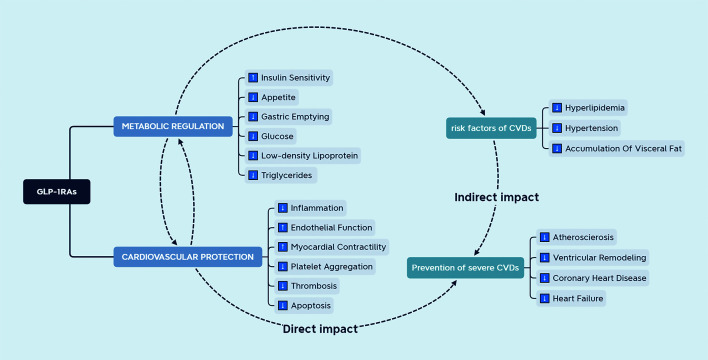
The effect of GLP-1RAs on the cardiovascular system. GLP-1RAs have direct and indirect effects on the cardiovascular system. Endocrine factors can ameliorate the risk factors for cardiovascular diseases.

Cardiovascular diseases (CVDs) are the main cause of premature death among Chinese individuals (107). In the cardiovascular system, GLP-1R is known to exist not only on cardiomyocytes and endothelial cells, but also in the autonomic nervous system, **Figure 3** suggests that GLP-1R may have direct and indirect effects on the heart and blood vessels. Moreover, it has been reported that GLP-1RAs increase the heart rate to a certain extent. However, this effect is soon weakened, not affecting the normal heart rate (108). The cardioprotective effects of GLP-1RAs include anti-inflammatory actions, reduction of myocardial ischemia injury, modifications in the synthesis and secretion of lipids, and improvement of endothelial dysfunction (109). A cardiovascular prognosis evaluation trial of semaglutide in the treatment of T2DM reported that administration of semaglutide reduced the cardiovascular prognostic risk (110). Nonetheless, the mechanism underlying the effects of GLP-1RAs in the cardiovascular system remain unclear. Many studies have shown that GLP-1RAs can treat certain CVDs, such as atherosclerosis and hypertension, and the suggested mechanism involves more than blood glucose control (111, 112). Therefore, the benefits of GLP-1 and GLP-1-based therapies for the cardiovascular system have attracted increased attention from clinicians.

## Effects on Atherosclerosis

Atherosclerosis (AS) is a fibrolipopathic formation on the arterial wall and a main cause of death worldwide. In particular, superimposed thrombosis is responsible for the most devastating consequences of AS, such as heart attacks and strokes. Elevated levels of plasma cholesterol constitute the most important factor in the development of AS (113). Briefly, the development of atherosclerotic lesions requires the action of low-density lipoprotein (LDL), which carries cholesterol through the blood (114). Over time, AS plaques form fibrous caps and accumulate calcium salts. Advanced AS plaques can often invade the arterial lumen, obstruct blood flow and cause tissue ischemia. In addition, following the collapse of an aneurysm, its contents are released in the blood flow, inducing thrombosis; the formed thrombus then blocks the lumen, also inducing tissue ischemia; usually with a more rapid onset (115). Inflammation is also known to be a key factor in the development of AS: the aggregation of white blood cells, mainly macrophages, is a significant feature of AS from its initial to its late stage (116).

A number of studies has found that semaglutide and liraglutide could reduce the levels of blood lipids and blood pressure (BP), hence contributing to the reduction of atherosclerosis and CVDs (117–119). Moreover, GLP-1RAs were reported to inhibit the development of AS in animal models. The anti-AS effects of GLP-1RAs include the regulation of inflammation (109), reduction of intima-media thickening (120), improvement of blood lipid profile (121), and regulation of endothelial dysfunction (122); however, the specific mechanism remains unclear.

As mentioned, GLP-1RAs can reduce the content of systemic inflammation markers. Importantly, controlling inflammation is believed to contribute in the prevention of cardiovascular diseases (123). In one study, liraglutide and semaglutide were reported to mitigate the development of plaque lesions by changing the inflammatory pathway in a mouse model of AS (124). Besides, GLP-1RAs might be driven by anti-inflammatory mechanisms to inhibit the formation of macrophage foam cells and thus slow down the pathological processes of AS (125, 126). Liraglutide was found to directly affect the formation of atherosclerotic lesions by tilting the macrophage population to the M-Φ-2 phenotype that is conducive to decomposition (127). Although this was demonstrated in animal experiments, GLP-1 was shown to exert acute anti-inflammatory and antioxidant effects on humans under specific conditions. Interestingly, these effects were found to not be simply regulated through the control of the levels of insulin (123). A number of studies has found that liraglutide prevented these AS effects of ox-LDL by preventing the inhibitory effect of the p53 protein on kruppel-like factor 2 (KLF2) of human aortic endothelial cells (114, 128). Of note, KLF2 has been shown to play an important role in protecting vascular endothelial cells from ox-LDL and oscillatory shear damage. As such, this finding provides a new option for preventing the development of AS. Another GLP-1 analogue, lixisenatide, was reported to prevent the acuteness of cardiovascular events in apolipoprotein E^−/−^insulin receptor substrate 2^+/−^ (APOE^−/−^irs2^+/−^) mice by reducing the size of AS plaques, increasing plaque stability, and promoting the conversion of macrophages to the anti-inflammatory M2 phenotype (129). In addition, liraglutide could also induce the cell cycle arrest of vascular smooth muscle cells through the AMPK pathway, thereby delaying the formation of AS (130). Although there is no GLP-1RA approved for the treatment of AS, these findings could provide novel approaches for the treatment of AS.

## Effects on Hypertension

Hypertension poses an increasingly serious public health problem worldwide. Hypertension can be divided into primary and secondary hypertension. Secondary hypertension is usually associated with an earlier age of onset, no family history, and a clear cause, such as renal or endocrine disorders, or iatrogenic triggers, such as the use of oral contraceptives (131). However, 90-95% of cases of hypertension are essential hypertension. Essential hypertension mostly occurs in middle-aged and elderly people, and is the result of the combination of lifestyle and genetic factors (132). Hypertension is the most common preventable risk factor for CVDs (including coronary heart disease, heart failure, stroke, myocardial infarction, atrial fibrillation, and peripheral artery disease), chronic kidney disease, and cognitive impairment, and also the main single factor for deaths and disability worldwide (133). The pathophysiological mechanism of hypertension is very complicated. The human body has a complex mechanism for maintaining the levels of blood pressure, including the renin-angiotensin-aldosterone system, natriuretic peptides, endothelial-sympathetic nervous system, and the immune system (134). Genetic factors are also implicated in the development of hypertension. For instance, the pathogenesis of essential hypertension involves multiple genes; certain allelic variants of several genes have been associated with an increased risk of essential hypertension, and almost all cases are known to be related to a familial history of hypertension (135). Finally, vasoactive substances produced by vascular endothelial cells are also important factors in the regulation of blood pressure.

A meta-analysis conducted by Wang et al. (136) showed that GLP-1RAa have the effect of lowering blood pressure. In particular, they found that the activation of GLP-1R promoted the secretion of atrial natriuretic peptide (ANP) and lowered blood pressure. In animal experiments, the GLP-1RA liraglutide was found to stimulate the secretion of ANP by increasing the levels of Epac2, which is a downstream target of GLP-1R signaling in cardiomyocytes (132), However, this finding seemed to contradict the results of clinical studies in which no increase was observed in the levels of ANP in healthy people and patients with T2MD. A study of patients with T2MD and congestive heart failure found that their levels of ANP were increased, whereas blood pressure was decreased, indicating that the GLP-1R-ANP axis is only active in patients with congestive heart failure (123). Despite the controversies about the expression of GLP-1R in vascular smooth muscle and endothelial cells, studies have shown that GLP-1RAs have anti-proliferative effects on these cells, and also reduced oxidative stress and increased the production of nitric oxide (NO) (137, 138). These effects have been confirmed in animal experiments, but not in humans, and the specific mechanism remains unknown. Nonetheless, these findings have already suggested the protective effect of GLP-1RAs on hypertension and atherosclerosis.

## Effects of Glucagon Like Peptide-1 Receptor Agonists on Endocrine Metabolism

**Figure 4:**
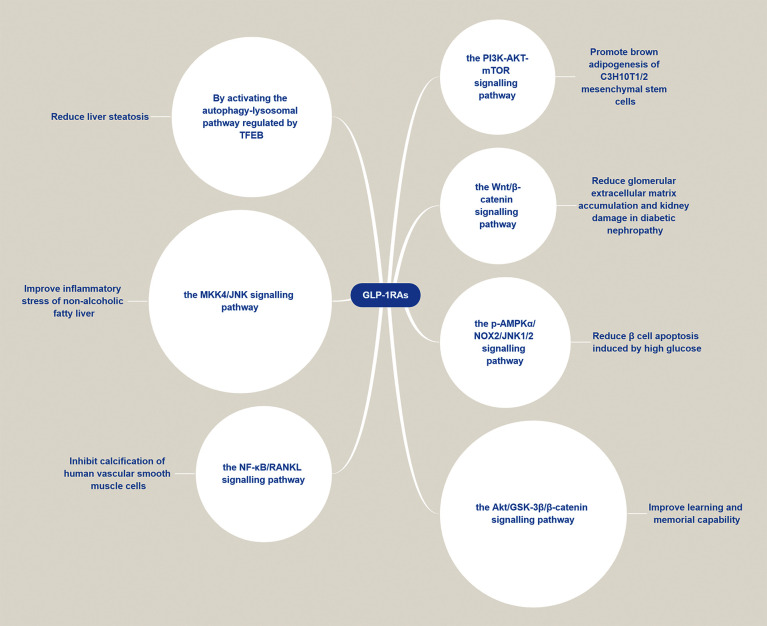
GLP-1RAs are involved in a variety of disease pathways. GLP-1RAs can activate several signaling pathways, such as the PI3K/AKT/mTOR, p-AMPKα/NOX2/JNK1/2, Wnt/β-catenin, Akt/GSK-3β/β-catenin, NF-κB/RANKL, and MKK4/JNK pathways thus exerting a therapeutic effect in different diseases.

Interestingly, GLP-1RAs not only exert a protective effect on the nervous and cardiovascular systems, but also exhibit a regulatory effect on metabolism. **Figure 4** illustrates the potential pathways of the function of GLP-1RAs against multiple diseases. For instance, GLP-1RAs can improve the levels of insulin, regulate the levels of sex hormone, improve the blood lipid profile, increase the levels of adiponectin, regulate autophagy, inhibit the production of liver glucose, reduce the liver fat content, as well as reduce the levels of plasma liver enzymes and liver steatosis. They can also be used to prevent and treat endocrine disorders or metabolic diseases such as polycystic ovary syndrome (PCOS), obesity, and non-alcoholic fatty liver disease (NAFLD) (135).

## Effects on Polycystic Ovary Syndrome

Polycystic ovary syndrome (PCOS), which is mainly manifested as sparse ovulation or non-ovulation, high levels of androgens, ovarian polycystic changes, obesity, and infertility, is the most common endocrine and metabolic disorder in women of childbearing age (139). When accompanied by metabolic disorders, such as IR and dyslipidaemia, it can lead to hyperlipidaemia, hypertension, metabolic syndrome and diabetes. In addition, skin problems such as hirsutism and androgenic alopecia cowing to the excessive levels of androgens might also occur (140–143). Although the pathogenesis of PCOS has not been fully elucidated, recent studies have indicated that excess levels of androgens might be the main pathogenic causative factor of PCOS (144–146). Patients with PCOS are frequently characterized by other symptoms, such as obesity and IR (147).

In women with PCOS, high concentrations of leptin have been reported to inhibit the expression of aromatase mRNA in granulosa cells, while the hyperandrogenic follicular environment in GCs was found to significantly reduce the expression of P450arom, which can convert androgens into estrogen, further increasing the levels of androgens and ultimately promoting follicular atresia (148–150). The high levels of free fatty acids (FFAs) are the main manifestation of obesity. Briefly, FFAs can reduce the tyrosine phosphorylation of insulin receptor substrate-1 by activating the serine/threonine kinase, thereby aggravating IR (151). Moreover IR in PCOS was shown to destroy the effect of gonadotropins, enhance the sensitivity of follicular membrane cells to the luteinizing hormone, and increase the expression of P450c17 and 3β-hydroxysteroid dehydrogenase type 2, leading to increased production of androgen (152). Concomitantly, the increased levels of insulin reduce the binding of liver sex hormone binding globulin SHBG to testosterone, leading to hyperandrogenism (153). Increased evidence have suggested that higher levels of androgen might cause follicular dysplasia, which is the main cause of insufficiency of ovulation (154).

Related studies have indicated a causal relationship between IR and all clinical symptoms of PCOS, with weight loss being shown to improve the reproductive function, hyperandrogenism and metabolism of women with PCOS (155, 156). Accordingly, GLP-1RAs have been increasingly used in the treatment of PCOS because of their ability to reduce weight and improve IR (155–159). Surprisingly, most studies have shown that GLP-1RAs not only can reduce the weight of patients with PCOS, but also have a positive effect on the levels of androgens (160, 161). However, there have been few studies on the potential mechanism of the action of GLP-1RAs in the treatment of PCOS. Some studies have suggested that the proliferation and apoptosis of granulosa cells (GCs) are the root causes of follicular development and atresia, with forkhead box protein O1 (FoxO1) playing an important role in promoting PCOS follicular atresia and GCs apoptosis (162, 163). The GLP-1/GLP-1R axis was demonstrated to enhance the activity of PCOS ovarian granulosa cells by partially modifying the FoxO1 phosphorylation sites, thereby promoting oocyte maturation (164). In addition, liraglutide was found to repair the cognitive impairment in a rat model of PCOS by inhibiting the overexpression of the Notch signaling pathway (165). Therefore, GLP-1RAs appear to have a beneficial role in the treatment of POCS; however, the specific mechanism of action needs to be studied in depth (166).

## Effects on Obesity

Obesity, as a chronic recurrent disease, is known to increase the risk of developing T2MD, hypertension, dyslipidemias, CVDs, NAFLD, and other diseases, leading to a poor quality of life and increased mortality (167). Obesity is considered to be related to increased appetite, changes in the levels of gastrointestinal hormones, increased fat mass, and disorders of satiety and satiation mechanisms (168). A recent trial on the treatment effect of semaglutide in people with obesity reported the significant effects of the long-acting GLP-1RA semaglutide on body weight and related phenotypes (169). Besides, it was found that semaglutide could regulate food preference, as well as reduce food intake and weight without reducing energy consumption (170).

A randomized controlled trial found that GLP-1 mimics, such as exenatide and liraglutide, had a significant weight loss effect on obese/overweight patients without diabetes (171). Likewise, once a week administration of efpeglenatide, a long acting GLP-1RA, significantly reduced the body weight in obese patients (172). Interestingly, supplementation of metformin with 1.5 mg dulaglutide per week in the treatment of T2MD in obese patients with severe impairment of glycosylated hemoglobin A1c (HbA1c) was reported to bring lasting benefits in the control of glucose metabolism and body weight (173). Moreover, there was no difference in weight changes between exenatide and abiglutide, and both were shown to exhibit remarkable weight loss effects (174). The GLP-1RAs-associated weight loss is thought to be achieved through a variety of mechanisms, including the delayed gastric emptying, increased satiety, increased resting energy expenditure, as well as the direct influence of the appetite center of the brain (175).

As GLP-1Rs are expressed in multiple brain regions, and GLP-1RAs such as liraglutide and lixisenatide can penetrate the blood-brain barrier, liraglutide and exendin-4 can be vagus-dependent or independent in the suppression of the food intake (176). Semaglutide is considered to control energy intake and reduce body weight probably by activating at discrete sites in the hypothalamus to reduce food craving, or by delaying the gastric emptying to affect appetite (119, 177, 178). In addition, GLP-1RAs could adjust the levels of the satiety signal in the arcuate nucleus in the hypothalamus, thereby increasing satiety and reducing caloric intake (179). Various studies have found that GLP-1RAs can play a role in controlling obesity through a variety of signaling pathways (180, 181). For example, they have been shown to activate the Wnt signaling pathway to promote adipocyte differentiation; while they could also rely on SIRT1 to mediate lipolysis and fatty acid oxidation in adipose tissues. When GLP-1RAs act on the gastrointestinal tract, they could extend the gastric emptying time. However, inhibiting gastric emptying mainly plays a role in reducing postprandial hyperglycaemia, and hence might not be the main mechanism of weight loss (176). In addition, GLP-1RAs could also increase energy consumption. Under the regulation of AMPK in the ventral medial hypothalamus, they could promote the conversion of visceral white adipose tissue (WAT) to brown adipose tissue (BAT), promoting thermogenesis of BAT and thus energy consumption (182). Finally, most patients could develop a tolerance mechanism to reduce or eliminate the GLP-1RAs-induced adverse reactions (182), thus allowing their use as drugs of choice for the treatment of obesity.

## Effects on Non-Alcoholic Fatty Liver Disease

Non-alcoholic fatty liver disease (NAFLD) is a metabolic stress liver injury closely related to IR and genetic variations. It is also known to be closely related to the T2MD metabolic syndrome, and the morbidity and mortality of CVDs (183, 184). About 10%-25% of patients with NAFLD will develop non-alcoholic steatohepatitis (NASH), which increases the risk of liver cirrhosis or liver cancer (185). A number of studies has suggested that IR might be the main mechanism of NAFLD (185, 186). Moreover, the increased morbidity and mortality in patients with NASH is known to be closely related to the severity of liver fibrosis. It has been shown that an increased percentage of fat, adipose tissue dysfunction, and IR lead to increased levels of FFAs and carbohydrates. These conditions of excessive lipid toxicity and metabolic load eventually lead to the accumulation of liver lipids, cell damage, inflammation, and fibrosis (187, 188).

A prospective study found (189) that 1 year after bariatric surgery, NASH was eliminated in 85% of patients, indicating that weight loss was closely related to the histological improvement of fatty liver. Oxidative stress also seems to be an important pathophysiological mechanism of NAFLD (190).

As mentioned above, GLP-1RAs possess multiple biological effects, such as lowering the levels of blood glucose and blood lipids, reducing body weight, and protecting the cardiovascular system. A previous study of liraglutide on mice fed a diet lacking methionine and choline also found that GLP-1RAs not only could improve IR, but also reduce harmful ceramide/nerve sheath types, and reduce NAFLD inflammation and fibrosis (191). Liraglutide could also improve hepatic steatosis and ballooning degeneration in patients with NASH (192). A 72-week, double-blind phase 2 trial showed that once-daily administration of semaglutide had a significant therapeutic effect on NASH (193). Long term use of beinaglutide, a recombinant human GLP-1(7-36) acid, was demonstrated to reduce the body weight and improve steatosis (194). Several studies found that GLP-1RAs might have direct effects on adipogenesis, lipotoxicity, fatty acid oxidation, cytokines related to hepatitis and fibrosis, and intestinal microbiota (193, 195). Therefore, GLP-1RAs are of great significance in the treatment of NAFLD.

As mentioned, GLP-1RAs can treat NAFLD through multiple mechanisms. Adiponectin was reported to reduce NAFLD inflammation through the AMPK-JNK/ErK1/2-NFκB/ROS signaling pathway, also exhibiting insulin sensitization and liver protection (196). A recent report covered that tirzepatide increased adiponectin levels and significantly decreased Nash related biomarkers, which was beneficial to improve NAFLD (197). In NAFLD mice, liraglutide could improve the hypoadiponectinemia-induced inflammatory stress by inhibiting the MKK4/JNK signaling pathway, inhibit liver adipogenesis by activating AMPK, and induce autophagy through the AMPK/mTOR pathway, hence improving the levels of liver lipids (23, 198). Further studies have shown that the SIRT1/HSF1/HSP pathway plays an important role in the exenatide-induced reduction of lipid-induced liver endoplasmic reticulum stress (199, 200). Concomitantly, autophagy was reported to also reduce the hepatocyte apoptosis caused by steatosis and endoplasmic reticulum stress, possibly through the GLP-1RA-stimulated GLP-1R-mediated activation of the EB transcription factor of the autophagy-lysosome pathway, thus reducing the accumulation of liver lipids (201). Therefore, GLP-1RAs appear to serve as novel candidates for the treatment of NAFLD.

## Effects of Glucagon Like Peptide-1 Receptor Agonists on Tumor Diseases

**Figure 5:**
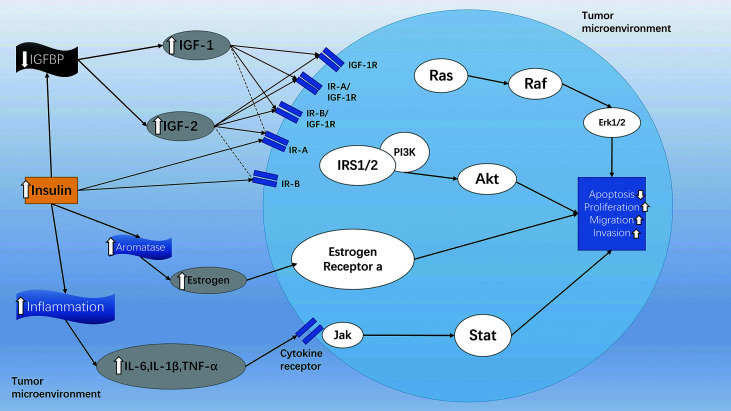
The effect of diabetes on tumor cell microenvironment and intracellular signaling transduction. Diabetic patients can increase their levels of insulin and stimulate the PI3K/Akt/mTOR, Ras/Raf/MAPK, and Jak/Stat signaling pathways in different ways, thereby inhibiting cancer cell apoptosis and promoting the spread, migration, and invasion of cancer cells. Solid arrows indicate strong affinity for the receptor, and dashed arrows represent weak affinity for the receptor. The figure is modified from Ref 204.

To date, a large number of epidemiological investigations have confirmed that the occurrence of endometrial cancer, hepatobiliary cancer, pancreatic cancer, breast cancer, prostate cancer, and colorectal cancer is positively correlated with T2MD (202, 203). In addition, hyperinsulinemia in diabetic patients seems to be the main reason for an increased risk of cancer (204). **Figure 5** illustrates the effect of diabetes on the tumor cell microenvironment and intracellular signaling transduction. The high levels of insulin have been shown to reduce the levels of the Insulin-like growth factor (IGF) binding protein, which can tightly bind to IGFs, resulting in excessive free IGF-1 in cells and tissues. In turn, the levels of IGF-1 have been shown to be associated with an increased risk of cancer (204, 205). Therefore, patients with T2MD are more likely to suffer from several malignancies compared with healthy individuals. However, selecting the right drug for the treatment of diabetic patients with malignancies is a difficult clinical decision. As mentioned, GLP-1RAs are increasingly used in the treatment of T2MD owing to their following advantages: lowering of the levels of blood glucose, reducing body weight, improving the function of islet, and exerting potential cardiovascular benefits (206). Whether GLP-1RAs could also affect the occurrence and development of tumors has been the focus of attention only in recent years.

A meta-analysis of clinical studies indicated that treatment with GLP-1RAs of obese patients with T2MD did not increase the risk of breast tumors, nor did it increase the risk of acute pancreatitis, pancreatic cancer, and overall tumor neoplasia (207–209). Furthermore, Gier et al. found that the use of GLP-1RAs in diabetic patients might not increase the risk of new thyroid tumors in patients with T2MD (208). Related studies have found that GLP-1RAs could limit the growth of prostate cancer by inhibiting the PI3K/AKT/mTOR and ERK/MAPK pathways, while could also limit the growth of pancreatic and prostate cancer cells by inhibiting the NF-kB pathway (210–212). Similarly, GLP-1RAs were also demonstrated to exert an inhibitory effect on the growth of breast and cervical cancer, implying the potential application of GLP-1RAs for the treatment of these cancer (10). Zhang et al. reported for the first time that liraglutide exhibited significant anti-proliferation and pro-apoptotic effects on gemcitabine-resistant human pancreatic cancer cells resistant to a variety of drugs, by regulating the NF-κB signaling pathway and downstream ATP-binding cassette subfamily G member 2 (ABCG2) (213, 214).

Likewise, Wenjing et al. found that exendin-4 could reduce the resistance of prostate cancer cells to enzalutamide by targeting the PI3K/AKT/mTOR pathway, while combined use of exendin-4 and enzalutamide could significantly inhibit the growth of prostate cancer cells (210). Therefore, a combination of GLP-1RAs with anticancer drugs could indirectly inhibit the migration, invasion, and growth of tumors by increasing the chemosensitivity of cancer cells. Interestingly, this approach seems to be more effective for the treatment of patients with advanced cancer (210, 215).

Liraglutide and exenatide were reported to induce apoptosis and autophagy through the AMPK signaling pathway, inhibiting the progression of endometrial cancer (216, 217). In addition, exenatide was shown to activate GLP-1R and inhibit the PI3K/AKT pathway to inhibit the growth, migration, and invasion of ovarian cancer cells, and promote cell apoptosis, thereby producing anticancer effects on diabetic patients with ovarian cancer (218). Besides, exenatide-4 has been found to inhibit the survival, migration, proliferation, and invasion of glioma cells in a GLP-1R/SIRT3 pathway dependent manner (219). The continuous in-depth research on the effects of GLP-1RAs on the occurrence and development of cancer in recent years have highlighted the increased potential for their use in the clinical treatment of cancer.

Unfortunately, the occurrence of some adverse events has caused deep concern about the long-term safety of GLP-1RAs. A population-based matched case (220) controlled study found that the treatment of patient with T2MD with exenatide might increase the chance of hospitalization for acute pancreatitis. In addition, Knapen (221) also found that the use of incretin drugs could increase the risk of pancreatic cancer by 1.7 times. However, because of the limited statistical power, insufficient follow-up time, and confusion about the severity of the disease, the researchers could not effectively conclude whether a direct relationship existed between the use of exenatide and the occurrence of acute pancreatitis. On the contrary, a systematic review on mortality, as well as cardiovascular and kidney statuses in patients with T2DM treated with GLP-1RAs concluded that there was no increase in the incidence of severe hypoglycaemia, pancreatitis, or pancreatic cancer (222). Cao (11) conducted a meta-analysis of 7 cardiovascular follow-up test results and discovered that the use of GLP-RAs was not correlated with the increased risk of pancreatic cancer. In contrast, they considered that obesity might be a risk factor for the development of pancreatitis and pancreatic cancer (223). Through rigorous scientific experiments, Zhao et al. showed that the activation of GLP-1R by liraglutide led to an anti-tumor effect on human pancreatic cancer *via* the inhibition of the PI3K/AKT pathway (224). In addition, a large-scale clinical study demonstrated that GLP-1 analogues could reduce the mortality of patients with prostate cancer and diabetes (19). Conclusively, with regard to patients with pancreatic cancer and diabetes, GLP-1RAs have the potential to treat pancreatic cancer while also controlling the levels of blood glucose (210, 224). A study on whether and how the activation of GLP-1R affects proliferation and apoptosis of human pancreatic cancer cells has demonstrated that GLP-1RAs might activate cAMP in a GLP-1R-dependent manner, subsequently inhibiting the AKT and ERK1/2 signaling pathways, hence inhibiting the growth of transplanted tumors *in vivo*, as well as inducing apoptosis and inhibiting the proliferation of a human pancreatic cancer cell line *in vitro (* 225). All the aforementioned results have suggested that GLP-1RAs are safe and beneficial for the treatment of pancreatitis and pancreatic cancer.

To date, no clear clinical evidence has been found to suggest the tumorigenic effect of GLP-1RAs (226), whereas plenty of studies have indicated that GLP-1RAs can inhibit the growth of ovarian, breast, prostate, and pancreatic cancer (227, 228). However, our understanding of the function of GLP-1RAs is not complete, and further research, including long-term and large clinical trials are warranted to properly evaluate the causal relationship between GLP-1RAs and the development and progression of various cancers.

## Conclusions and Future Perspectives

Overall, GLP-1RAs are considered novel blood glucose lowering drugs used in the treatment of T2MD (229), owing to their advantages of lowering blood pressure, lowering the levels of fasting blood glucose, lowering HbA1c, inducing weight loss, and being associated with a low incidence of hypoglycemia. In addition, GLP-1RAs are valuable in the treatment and prevention of diseases of the nervous and cardiovascular system, endocrine disorders, or metabolic diseases due to their neuroprotective, cardiovascular protective, and metabolic regulatory effects. However, there are still many controversies regarding the tolerability and adverse reactions of GLP-1RAs in the treatment of diabetes. Almost all diseases and drug treatments are inevitably associated with side effects, such as nausea and vomiting. Surprisingly though, a second-generation GLP-1RA conjugate composed of vitamin B12 and exendin-4 was reported to reduce the adverse reactions, exhibiting improved tolerance (230). Another imminent problem is that the GLP-1RAs that are currently used in the clinical setting are biological macromolecular peptide preparations, such as exenatide and liraglutide, with high production costs that limit their clinical application (231). One obvious advantage of the small molecule agonists in the replacement of peptide GLP-1 analogues is that they could be administered orally, thus avoiding the discomfort of subcutaneous injections and increasing the compliance of patients. Nowadays, LY3502790 and PF-06882961 (231–233), a class of oral non-peptide small molecule GLP-1RAs with preferable efficacy and anti-diabetes potential, are at the stage of pre-clinical research. Such developments are expected to greatly increase the confidence of patients regarding the improved efficiency and tolerance of GLP-1RAs. Therefore, we believe that the discovery of low-cost oral GLP-1RA analogues or synergists and the continuous elucidation of the possible mechanism of action of GLP-1 are of great significance for the expansion of the field of disease treatment.

## Outstanding Questions

GLP-1RA is a new type of drug for the treatment of T2MD. Recent studies have found that GLP-1RAs have therapeutic effects on neurological, cardiovascular, endocrine and metabolic diseases. More and more studies are looking for oral GLP-1RA analogs or synergists to enhance its therapeutic effect. This article systematically summarizes the extra-pancreatic effects of GLP-1RAs, and discusses the therapeutic effects and possible mechanisms of GLP-1RAs on various diseases. We believe that this article is of great significance to the research field and can provide ideas and directions for follow-up research.

## Search Strategy and Selection Criteria

Data for this Review were identified by searches of MEDLINE, Current Contents, PubMed, and references from relevant articles using the search terms “GLP-1”, “cardiovascular”, “neurological”, “endocrine” and “cancer”. Abstracts and reports from meetings were included only when they related directly to previously published work. Only articles published in English between 1980 and 2021 were included.

## Author Contributions

XZ and MW mainly wrote and revised the manuscripts, and constructed the framework of the manuscript. LC and CF provided constructive opinions on the formation of the manuscript. ZL, ZW, HX, and YL participated in the drawing of manuscript pictures and the investigation and sorting of documents. YZ participated in topic design, manuscript writing, manuscript editing and providing instructional support. All authors contributed to the article and approved the submitted version.

## Funding

This work was supported by NSFC (81770776; 81973378, 82073909), Cultivate Scientific Research Excellence Programs of Higher Education Institutions in Shanxi (2019KJ022).This research project was supported by FSKSC and 1331KSC.

## Conflict of Interest

The authors declare that the research was conducted in the absence of any commercial or financial relationships that could be construed as a potential conflict of interest.

## Publisher’s Note

All claims expressed in this article are solely those of the authors and do not necessarily represent those of their affiliated organizations, or those of the publisher, the editors and the reviewers. Any product that may be evaluated in this article, or claim that may be made by its manufacturer, is not guaranteed or endorsed by the publisher.
